# The title: serum neutrophil Gelatinase-associated Lipocalin at 3 hours after return of spontaneous circulation in patients with cardiac arrest and therapeutic hypothermia: early predictor of acute kidney injury

**DOI:** 10.1186/s12882-020-02054-7

**Published:** 2020-09-07

**Authors:** Yoon Hee Choi, Dong Hoon Lee, Jae Hee Lee

**Affiliations:** 1 Department of Emergency Medicine, College of Medicine, Ewha Womans University Mokdong Hospital, 1071 Anyangcheon-ro, Yangcheon-gu, Seoul, 07985 South Korea; 2 Department of Emergency Medicine, Chung-Ang University, College of Medicine, Seoul, South Korea

**Keywords:** Acute kidney injury, Out-of-hospital cardiac arrest, Targeted temperature management, Serum neutrophil gelatinase-associated lipocalin

## Abstract

**Background:**

Serum neutrophil gelatinase-associated lipocalin (NGAL) could be used as a predictive marker of acute kidney injury (AKI) in patients with return of spontaneous circulation (ROSC) after out-of-hospital cardiac arrest (OHCA) who are managed with targeted temperature management (TTM). However, the NGAL measurement timepoints vary from immediately after ROSC to several days later. The primary objective of this study was to determine an association between AKI and NGAL, both immediately (ROSC-NGAL) and 3 h after ROSC (3 h-NGAL), in OHCA patients with TTM. The secondary objective was to ascertain the association between NGAL levels in the early post-ROSC phase and the neurologic outcomes at discharge.

**Methods:**

This prospective observational study was conducted between January 2016 and December 2018 and enrolled adult OHCA patients (≥18 years) with TTM after ROSC. The serum NGAL level was measured both immediately and 3 h after ROSC. Univariate and multivariate analyses were performed to identify the associations between AKI, poor neurologic outcome, and NGAL.

**Results:**

Among 861 OHCA patients, 89 patients were enrolled. AKI occurred in 48 (55.1%) patients. On multivariate logistic regression analysis, 3 h-NGAL was significantly associated with AKI (odds ratio [OR] 1.022; 95% confidence interval [CI] 1.009–1.035; *p* = 0.001). The area under the receiver operating characteristic curve of 3 h-NGAL for AKI was 0.910 (95% CI 0.830–0.960), and a cut-off value of 178 ng/mL was identified. Both ROSC-NGAL and 3 h-NGAL were not significantly associated with poor neurologic outcome on multivariate logistic regression analysis (ROSC-NGAL; OR 1.017; 95% CI 0.998–1.036; *p* = 0.084, 3 h-NGAL; OR 0.997; 95% CI 0.992–1.001; *p* = 0.113).

**Conclusions:**

The serum NGAL concentration measured 3 h after ROSC is an excellent early predictive marker for AKI in OHCA patients treated with TTM. Future research is needed to identify the optimal measurement timepoint to establish NGAL as a predictor of neurologic outcome and to validate the findings of this research.

## Background

The post-cardiac arrest syndrome (PCAS) develops in cardiac arrest patients after the return of spontaneous circulation (ROSC) and comprises anoxic brain injury, post-cardiac arrest myocardial dysfunction, systemic ischemia–reperfusion response, and persistent precipitating pathology [1]. The ischemia–reperfusion response of PCAS could injure various organs, including the kidneys, which in turn could lead to AKI [2]. AKI occurs in approximately half of patients with PCAS and is associated with poor clinical outcome [3–7]. The serum creatinine level is the gold standard diagnostic criterion for AKI. However, one of the limitations with the use of the serum creatinine level as a diagnostic criterion is its inability to indicate mild/early-stage renal injury [8]. Neutrophil gelatinase–associated lipocalin (NGAL), which is one of the most researched biologic markers of AKI, have clinical utility as an early marker of AKI [9]. The evidence from the previous research indicates that NGAL could facilitate an AKI diagnosis in adult critically ill patients 48 h earlier than the Risk, Injury, and Failure, and Loss, and End-stage kidney disease, or RIFLE, criteria [10]. NGAL was thought to be produced in the kidneys [11]; however, a recent study has reported different results. In a basic research study by Skrypnyk et al., interleukin-6 (IL-6) was shown to mediate hepatic NGAL production in AKI in a mice. Those authors reported that hepatocytes are the primary source of plasma and urine NGAL during AKI [12]. However, AKI is a highly complex systemic disorder, and there are limitations with regard to the generalizability of the abovementioned results.

Several reports of the positive association between AKI and NGAL in post-cardiac arrest patients indicate that NGAL could be used as a predictive marker of AKI; however, in those studies, the timepoint of NGAL measurement varied from immediately after ROSC to several days later [13–15]. If the NGAL level in early-stage of ROSC is correlated with the occurrence of AKI, then, this would enable the prediction of AKI and the early initiation of appropriate management.

This study aimed to evaluate the correlation between NGAL and AKI to evaluate the use of NGAL as a predictive marker for AKI in patients with PCAS. The primary objective of this study was to determine an association between AKI and NGAL, both immediately and 3 h after ROSC, in out-of-hospital cardiac arrest (OHCA) patients who underwent targeted temperature management (TTM) after ROSC. The secondary objective was to ascertain the association between NGAL levels in the early post-ROSC phase and the neurologic outcomes at discharge.

## Methods

### Study setting and data collection

This prospective observational study was conducted at single tertiary hospital in Seoul, South Korea between January 2016 and December 2018. The target study population comprised all adult OHCA patients (age ≥ 18 years) who underwent TTM after ROSC. Patients with active intracranial bleeding, a do-not-resuscitate order, underlying disease with life expectancy < 6 months, pre-arrest cerebral performance category of 3 or 4, body temperature < 30° (the abovementioned contraindications for TTM [16]), end-stage renal disease, and missing data on NGAL measurements were excluded.

All study participants underwent post-cardiac arrest care and TTM in accordance with standardised institutional protocol. Baseline patient information and clinical data were collected through a chart review of the electronic medical records, whereas the data on the 1- and 6-month post-discharge survival were obtained by telephonic follow-up and accordingly recorded. If the patient died during the follow-up period, the date of death was recorded.

This study was approved by the institutional review board of Ewha Womans University Mokdong Hospital.

### Outcome measures

The primary outcome was the occurrence of AKI during hospitalisation. AKI was diagnosed on the basis of the Kidney Disease Improving Global Outcomes (KDIGO) guidelines, by using the serum creatinine level and urinary output [17]. Thus, AKI was defined based on any of the following: increase in serum creatinine (SCr) by ≥0.3 mg/dL (26.5 μmol/L) within 48 h; increase in SCr to ≥1.5 times the baseline, which is known or presumed to have occurred within the past 7 days; or urinary volume < 0.5 mL/kg/h for 6 h. The severity of AKI was determined according to the following criteria: Stage 1, increase in SCr up to 1.5 to 1.9 times the baseline value or increase in SCr ≥0.3 mg/dL (≥26.5 μmol/L) or urine output < 0.5 mL/kg/h for 6–12 h; Stage 2, increase in SCr 2.0 to 2.9 times the baseline value or urine output < 0.5 mL/kg/h for ≥12 h; Stage 3, increase in SCr to 3.0 times the baseline value or SCr ≥4.0 mg/dL (≥353.6 μmol/L) or initiation of renal replacement therapy or decreased eGFR to < 35 mL/min/1.73 m^2^ in patients younger than 18 years or with urine output < 0.3 mL/kg/h for ≥24 h or anuria for ≥12 h. For patients who were treated at the study centre before the cardiac arrest event or with information available on the creatinine level through medical records from another hospital, the previously recorded creatinine level was used as the baseline value. In patients without a previous creatinine level, the lowest value from tests performed within 24 h after ROSC was used as the baseline value. Creatinine was measured immediately after ROSC and again at 3 h after ROSC. After being admitted to the intensive care unit, laboratory tests were conducted daily or two times a day. The secondary outcome was the neurologic outcome at discharge, which was measured by using the Cerebral Performance Category (CPC) score that comprises five categories: good recovery (CPC 1), moderated disability (CPC 2), severe disability (CPC 3), vegetative state (CPC 4), and brain death or death (CPC 5) [18]. In this study, good neurologic outcome (GNO) was categorised as CPC 1 and 2 and poor neurologic outcome (PNO) as CPC 3–5.

The NGAL, which is an acute-phase protein after ischemic or nephrotoxic AKI, can be measured in urine or serum samples [19], and the serum NGAL can be detected as early as 2–4 h after kidney injury [19, 20]. Therefore, the present study used two measurements of serum NGAL – one taken immediately after ROSC (ROSC-NGAL) and another reading at 3 h after ROSC (3 h-NGAL) – to determine the usefulness of NGAL as an early predictor of AKI.

Data on baseline patient characteristics, including sex, age, and medical history, were collected. The following factors were identified with regard to the cardiac arrest event: initial rhythm, witnessed cardiac arrest, bystander cardiopulmonary resuscitation (CPR), time from emergency medical service (EMS) activation to arrival of EMS, time from EMS activation to first defibrillation, time to ROSC, and dose of epinephrine used during CPR.

To determine the post-ROSC patient condition, we collected information on the following factors: continuous renal replacement therapy, coronary angiography, duration of TTM and targeted body temperature, survival at discharge, CPC at discharge, and 1- and 6-month survival.

### Statistical analysis

Data are expressed as median with interquartile ranges for continuous data with non-normal distribution, and as the number with percentages for categorical variables. The study population was divided into two subgroups based on AKI occurrence and neurologic outcome at discharge, and intergroup comparisons of general characteristics and clinical findings were undertaken. For items that required statistical verification, the Mann–Whitney *U* test was used for continuous variables, and the chi-square or Fisher’s exact test was used for categorical variables. Binary logistic regression analysis was used to assess the predictor variables that were identified on univariate analyses. Odds ratios (ORs) and 95% confidence intervals (CIs) were computed from the estimated coefficients in the regression model. All statistical analyses were conducted in SPSS version 21.0 for Windows (SPSS Inc., Chicago, IL, USA). The adjusted ORs and 95% CIs were obtained from multivariate analyses. Furthermore, to determine the predictive performance of a significant variable for AKI, a receiver operating characteristic (ROC) curve analysis was created by using MedCalc Statistical Software version 19 (MedCalc Software BVBA, Ostend, Belgium). Moreover, the areas under the ROC curve (AUROCs) and 95% CIs were calculated, and the AUROCs were compared by DeLong’s method [21]. A two-tailed *p*-value of < 0.05 was considered statistically significant.

## Results

**Fig. 1:**
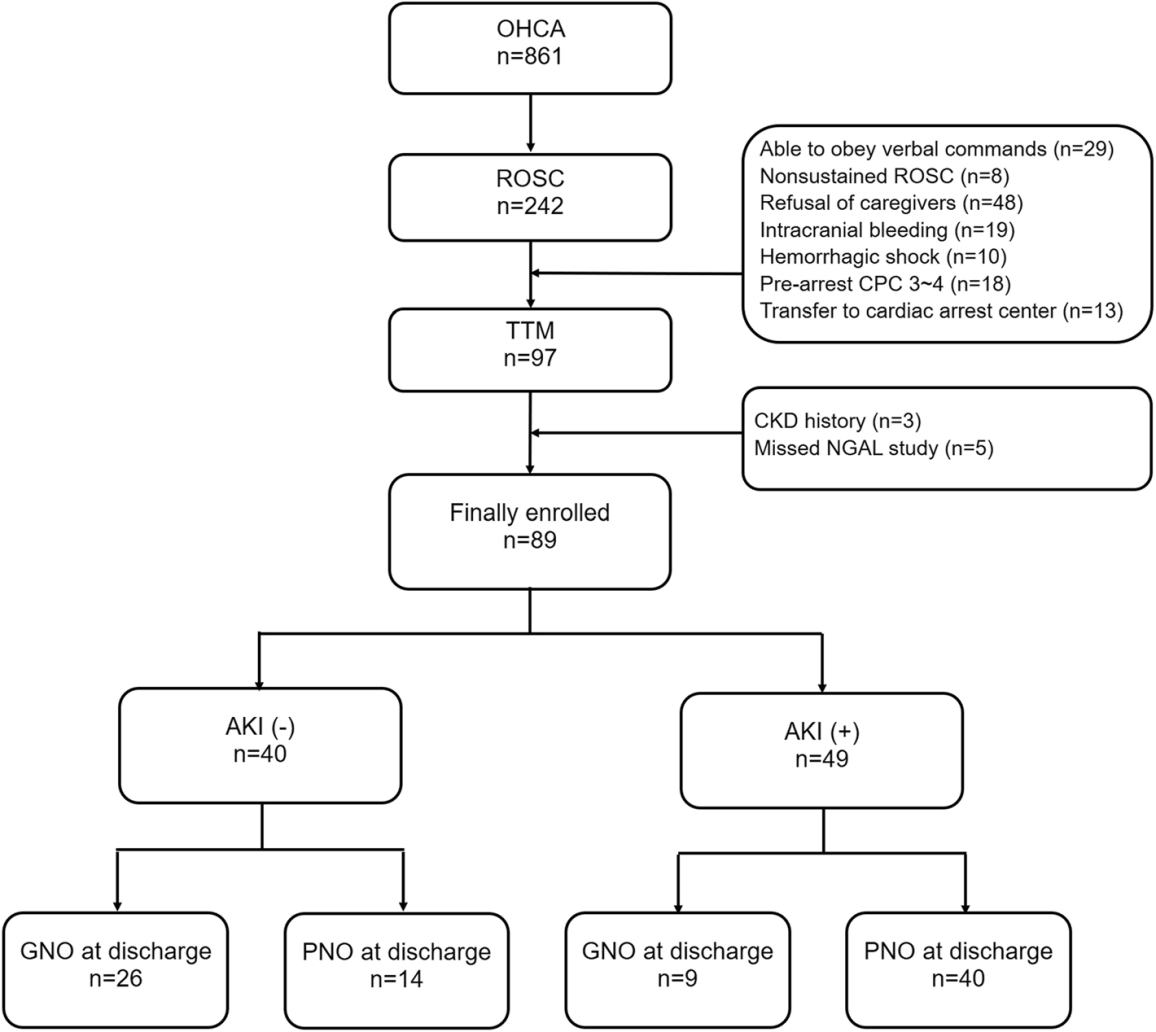
Flow chart of the study population. *OHCA* out-of-hospital cardiac arrest: *ROSC* return of spontaneous circulation: *TTM* targeted temperature management: *CKD* chronic kidney disease: *NGAL* neutrophil gelatinase-associated lipocalin: *AKI* acute kidney injury: *GNO* good neurologic outcome: *PNO* poor neurologic outcome

During the study period, a total of 861 OHCA patients were admitted to the emergency room, and 97 of them received TTM. After excluding patients without NGAL measurement values and patients with chronic kidney disease, 89 patients (mean age 53.8 years; 63 males [70.8%]) were included in the final study population (Fig. 1).

### General characteristics of study participants

The general characteristics (Table 1) were compared between the two study groups, which were stratified by the presence or absence of AKI (AKI (+) group vs AKI (−) group). The AKI (+) group included 48 patients (55.1%). The AKI (−) group included 40 patients (44.9%). There were no significant intergroup differences in the sex distribution, mean age, and presence of underlying diseases. With regard to the initial rhythm, the AKI (+) group showed a significantly higher asystole rate (51.1%), whereas the AKI (−) group showed a higher incidence of ventricular fibrillation (Vf; 66.7%) and pulseless electrical activity (PEA; 23.1%). The rate of witnessed cardiac arrest was higher in the AKI (−) than in the AKI (+) group (92.5% vs 59.2%), whereas significantly higher epinephrine doses were used in the AKI (+) group during CPR. Furthermore, there were no significant intergroup differences with regard to bystander CPR, time from EMS activation to EMS arrival, time from EMS activation to first defibrillation, and time until ROSC. The general characteristics were analysed according to the AKI stage (Additional file 1). In the AKI (+) group, the proportion of stages 1, 2, and 3 AKI was 23.6, 13.5, and 18.0% respectively. Compared to the AKI (+) vs AKI (−) group analysis, a similar trend was seen in the AKI stage analysis except for the medical history of diabetes mellitus. With regard to diabetes mellitus, there was no significant difference in the distribution of the incidence in the AKI (+) and AKI (−) groups (*p*-value 0.202), although there was a significant difference in the distribution of groups by the AKI stages (*p* = 0.025, AKI (−) group, 12.5%; AKI Stage 1, 9.5%; AKI Stage 2, 41.7%: and AKI Stage 3, 37.5%). There was no significant different in baseline creatinine values among the groups stratified by the AKI stage. The peak creatinine level was significantly different according to the AKI stages: 1.35 (95% CI 1.04–1.26) for Stage 1, 2.11 (95% CI 1.99–2.38) for Stage 2, and 4.66 (95% CI 3.70–7.38) for Stage 3.

**Table 1:** General characteristics of study patients

Characteristics	Total		Acute kidney injury					Neurologic outcome at discharge				
			(−)		(+)		*p-*value	Good		Poor		*p-*value
Number of patients	89	(100.0)	40	(44.9)	49	(55.1)		35	(39.3)	54	(60.7)	
Sex							0.430					0.124
Male	63	(70.8)	30	(75.0)	33	(67.3)		28	(80.0)	35	(64.8)	
Female	26	(29.2)	10	(25.0)	16	(32.7)		7	(20.0)	19	(35.2)	
Age (years)	56.0	(43.5–66.0)	53.0	(43.0–62.0)	58.0	(44.5–67.0)	0.358	55.0	(43.0–62.0)	56.0	(44.8–68.0)	0.245
Medical history												
HTN	28	(31.5)	11	(27.5)	17	(34.7)	0.467	13	(37.1)	15	(27.8)	0.353
DM	18	(20.2)	5	(12.5)	13	(26.5)	0.101	7	(20.0)	11	(20.4)	0.966
HF	2	(2.2)	1	(2.5)	1	(2.0)	1.000	1	(2.9)	1	(1.9)	1.000
Initial rhythm by EMS or hospital							**< 0.001**					**< 0.001**
Vf	40	(44.9)	26	(65.0)	14	(28.6)		26	(74.3)	14	(25.9)	
PEA	18	(20.2)	9	(22.5)	9	(18.4)		5	(14.3)	13	(24.1)	
Asystol	28	(31.5)	4	(10.0)	24	(49.0)		3	(8.6)	25	(46.3)	
Unknown	3	(3.4)	1	(2.5)	2	(4.1)		1	(2.9)	2	(3.7)	
Witness cardiac arrest	66	(74.2)	37	(92.5)	29	(59.2)	**< 0.001**	32	(91.4)	34	(63.0)	**0.003**
Bystander CPR	49	(55.1)	25	(62.5)	24	(49.0)	0.202	20	(57.1)	29	(53.7)	0.750
EMS activation to EMS arrival (min)	7.0	(5.6–10.0)	7.0	(6.0–9.0)	7.0	(5.0–10.0)	0.922	6.0	(5.0–10.0)	8.0	(6.0–10.0)	0.305
EMS activation to first defibrillation (min)	7.5	(6.0–12.0)	7.5	(6.0–10.0)	7.5	(5.6–12.3)	1.000	7.0	(6.0–10.0)	9.0	(6.0–12.0)	0.389
Time to ROSC (min)	25.0	(13.8–35.0)	19.0	(11.5–33.5)	27.0	(14.0–36.5)	0.410	16.5	(10.5–29.5)	30.5	(14.8–37.3)	**0.007**
Epinephrine dose during CPR	1.0	(0.0–3.0)	0.0	(0.0–1.0)	3.0	(1.0–4.0)	**< 0.001**	0.0	(0.0–0.0)	2.5	(1.0–4.0)	**< 0.001**

Quantitative data are expressed as median (interquartile range), categorical data are presented as number of subjects (percentages). Mann-Whitney *U* test was used for continuous variable analysis, while chi-squared test or Fisher’s exact test were used for categorical variable analysis as appropriate

*HTN* hypertension, *DM* diabetes mellitus, *HF* heart failure, *Vf* ventricular fibrillation, *PEA* pulseless electrical activity, *CPR* cardiopulmonary resuscitation, *EMS* emergency medical system, *ROSC* return of spontaneous circulation

The intergroup differences in neurologic outcomes at discharge (GNO group vs PNO group) showed that the PNO group (CPC 3–5) included 54 patients (60.7%). There were no significant between-group differences in the sex distribution, mean age, and presence of underlying diseases in the GNO and PNO groups. Similar to the AKI (+) group, the PNO group showed a significantly higher asystole rate (48.1%), whereas the GNO group showed higher rates of Vf (26.9%) and PEA (25.0%). The frequency of witnessed cardiac arrest was higher in the GNO group (91.4% vs 63.0%), whereas time to ROSC was longer in the PNO group (30.5 [14.0–37.3] min vs 16.5 [10.5–29.5] min). The epinephrine dose used in CPR was significantly higher in the PNO group. However, there were no significant differences in bystander CPR, time from EMS activation to EMS arrival, and time from EMS activation to first defibrillation.

### Comparison of clinical characteristics according to AKI occurrence and neurological outcome

**Fig. 2:**
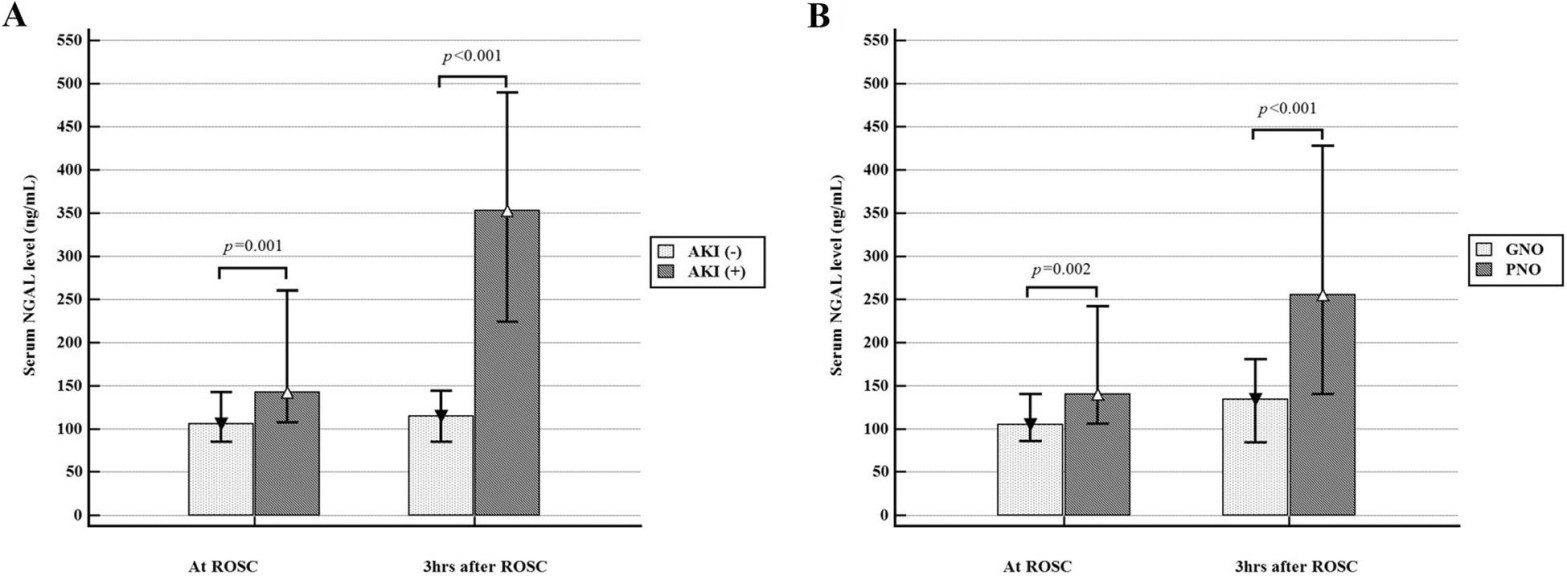
**a.** Serum NGAL level at immediately and 3 h after ROSC according to acute kidney injury development. **b.** Serum NGAL level at immediately and 3 h after ROSC according to neurologic outcome at discharge. *NGAL* neutrophil gelatinase-associated lipocalin*: ROSC* return of spontaneous circulation: *AKI* acute kidney injury *GNO* good neurologic outcome: *PNO* poor neurologic outcome

The clinical characteristics were compared according to AKI occurrence and neurological outcome (Table 2). The ROSC-NGAL and 3 h-NGAL were significantly higher in the AKI (+) group. In addition, the AKI (−) group showed a significantly higher percentage of survival at discharge (85.0% vs 28.6%). The percentage of poor outcome based on the CPC score was higher in the AKI (+) group (81.6% vs 35.0%). Furthermore, the rates of 1- and 6-month survival were significantly higher in the AKI (−) group (1-month survival: 82.5% vs 28.6%; 6-month survival: 82.5% vs 29.2%; Table 2 and Fig. 2). Furthermore, the clinical characteristics according to the AKI stage were analysed (Additional file 1). Compared to the AKI (+) vs AKI (−) group analysis, a similar trend was observed in the sub-analysis by the AKI stage.

**Table 2:** Clinical characteristics after return of spontaneous circulation

	Total		Acute kidney injury					Neurologic outcome at discharge				
			(−)		(+)		*p*-value	Good		Poor		*p*-value
NGAL at ROSC	124.0	(96.0–186.0)	105.5	(83.3–143.3)	142.0	(107.0–263.0)	**0.001**	105.0	(84.0–141.0)	140.0	(140.6–245.8)	**0.002**
NGAL at 3 h after ROSC	181.0	(115.0–381.0)	115.0	(84.3–145.5)	353.0	(223.0–510.0)	**< 0.001**	134.0	(84.0–181.0)	255.5	(139.5–434.8)	**< 0.001**
CRRT	11	(14.7)	0	(0.0)	11	(23.4)	**0.005**	1	(3.6)	10	(23.1)	**0.045**
CAG	40	(44.9)	25	(62.5)	15	(30.6)	**0.003**	30	(85.7)	10	(18.5)	**< 0.001**
Target temperature							0.586					0.559
33 °C	86	(96.6)	38	(95.0)	48	(98.0)		33	(94.3)	53	(98.1)	
< 36 °C	3	(3.4)	2	(5.0)	1	(2.0)		2	(5.7)	1	(1.9)	
TTM duration							0.624					1.000
24 h	85	(95.5)	39	(97.5)	46	(93.9)		34	(97.1)	51	(94.4)	
48 h	4	(4.5)	1	(2.5)	3	(6.1)		1	(2.9)	3	(5.6)	
Survival discharge	48	(53.9)	34	(85.0)	14	(28.6)	**< 0.001**	35	(100.0)	13	(24.1)	**< 0.001**
CPC at discharge							**< 0.001**					
Good (1,2)	35	(39.3)	26	(65.0)	9	(18.4)						
Poor (3,4,5)	54	(60.7)	14	(35.0)	40	(81.6)						
Survival at 1 month	47	(52.8)	33	(82.5)	14	(28.6)	**< 0.001**	35	(100.0)	12	(22.2)	**< 0.001**
Survival at 6 months	47	(53.4)	33	(82.5)	14	(29.2)	**< 0.001**	35	(100.0)	12	(22.6)	**< 0.001**

Quantitative data are expressed as median (interquartile range), categorical data are presented as number of subjects (percentages). Mann-Whitney *U* test was used for continuous variable analysis, while chi-squared test or Fisher’s exact test were used for categorical variable analysis as appropriate

*NGAL* neutrophil gelatinase-associated lipocalin, *ROSC* return of spontaneous circulation, *CRRT* continuous renal replacement therapy, *CAG* coronary angiography, *TTM* targeted temperature management, *CPC* cerebral performance category

Intergroup comparison of the ROSC-NGAL and 3 h-NGAL levels in the groups stratified by the neurologic outcome at discharge showed significantly higher levels of NGAL in the PNO group. The percentage of patients who needed continuous renal replacement therapy was significantly higher in the PNO group (23.1% vs 3.6%), whereas a significantly higher percentage of patients in the GNO group underwent coronary angiography (85.7% vs 18.5%; Table 2 and Fig. 2).

### Univariate and multivariate logistic regression analysis for AKI

To examine the predictors of AKI, we undertook logistic regression analysis to identify the factors that significantly differed between the AKI (+) and AKI (−) groups. The results of multivariate logistic regression analysis showed that unwitnessed cardiac arrest (OR 8.274; 95% CI 1.287–53.18) and 3 h-NGAL (OR 1.022; 95% CI 1.009–1.035) were significantly associated with AKI (Table 3).

**Table 3:** Univariable and multivariable logistic regression analysis for acute kidney injury and poor neurologic outcome at discharge

Variables	Univariable			Multivariable		
	OR	(95% CI)	*p*-value	OR	(95% CI)	*p*-value
Acute kidney injury						
Non-shockable rhythm	4.714	(1.891–11.750)	0.001	2.963	(0.546–16.090)	0.208
Witness cardiac arrest: No	8.506	(2.301–31.437)	0.001	8.274	(1.287–53.18)	**0.026**
Epinephrine dose during CPR	1.922	(1.362–2.713)	< 0.001	1.282	(0.830–1.979)	0.263
NGAL at ROSC	1.009	(1.002–1.017)	0.014	0.988	(0.969–1.008)	0.230
NGAL at 3 h after ROSC	1.019	(1.011–1.028)	< 0.001	1.022	(1.009–1.035)	**0.001**
Poor neurologic outcome at discharge						
Non-shockable rhythm	8.821	(3.240–24.020)	< 0.001	3.005	(0.627–14.396)	0.169
Witness cardiac arrest: No	6.275	(1.700–23.161)	0.006	8.357	(1.211–57.654)	**0.031**
Time to ROSC	1.057	(1.014–1.102)	0.008	1.021	(0.962–1.084)	0.495
Epinephrine dose during CPR	3.833	(2.056–7.146)	< 0.001	3.348	(1.465–7.652)	**0.004**
NGAL at ROSC	1.011	(1.002–1.019)	0.014	1.017	(0.998–1.036)	0.084
NGAL at 3 h after ROSC	1.004	(1.001–1.007)	0.022	0.997	(0.992–1.001)	0.113

*CPR* cardiopulmonary resuscitation, *NGAL* neutrophil gelatinase-associated lipocalin, *ROSC* return of spontaneous circulation

To examine the predictors of poor neurologic outcome at discharge, we conducted a logistic regression analysis to identify factors that showed significant differences between the good and poor outcome groups. Multivariate logistic regression analysis showed that unwitnessed cardiac arrest (OR 8.357; 95% CI 1.211–57.654) and the higher dose of epinephrine used during CPR (OR 3.348; 95% CI 1.465–7.652) were significantly associated with poor neurologic outcome (Table 3).

### ROC curve and cut-off value of NGAL at 3 hours after ROSC for AKI

**Fig. 3:**
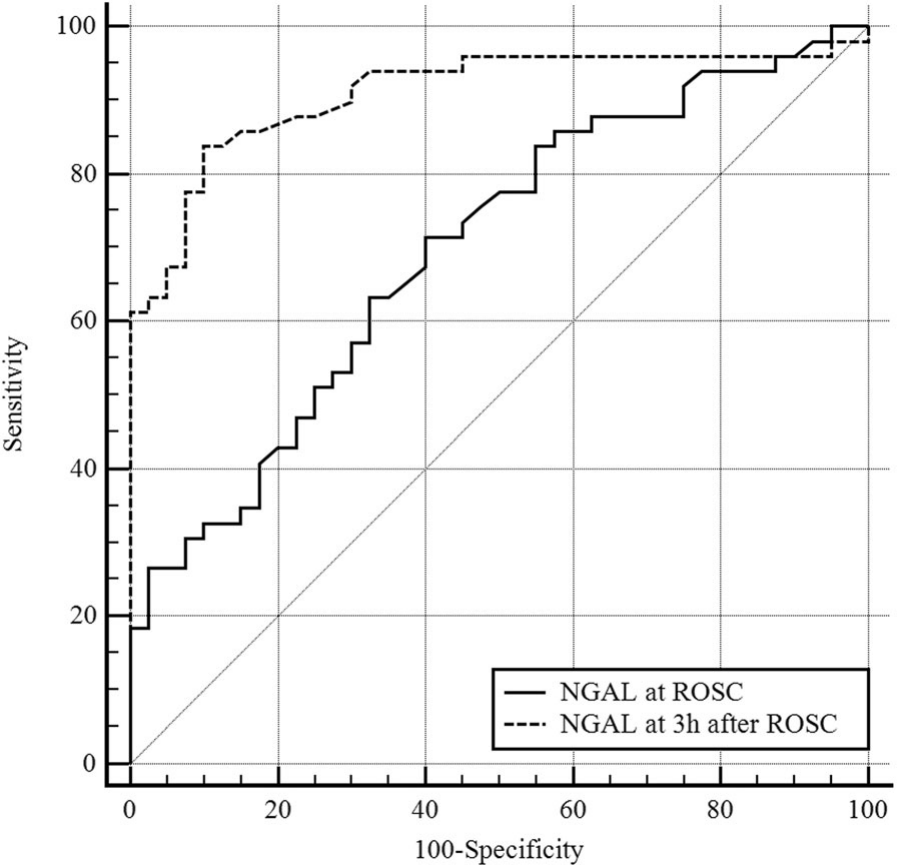
ROC curve of NGAL for acute kidney injury development. *NGAL* neutrophil gelatinase-associated lipocalin*: ROSC* return of spontaneous circulation

A ROC curve analysis was conducted to verify the clinical usefulness of 3 h-NGAL as a predictor of AKI. The AUROC of 3 h-NGAL for AKI was 0.910 (95% CI 0.830–0.960), whereas the cut-off value was 178 ng/mL. Therefore, the sensitivity, specificity, positive likelihood ratio, and negative likelihood ratio for AKI were 83.67 (95% CI 70.3–92.7), 90.00 (95% CI 76.3–97.2), 8.37 (95% CI 3.3–21.4), and 0.18 (95% CI 0.10–0.3), respectively. The AUROC of ROSC-NGAL was 0.698 (95% CI 0.591–0.791), which was significantly lower than that of 3 h-NGAL (*p*-value = 0.0001; Fig. 3).

## Discussion

This study aimed to compare and analyse the NGAL levels at ROSC and 3 h post ROSC in AKI among patients who underwent TTM, and to evaluate the clinical utility of NGAL as a predictive marker for AKI. Moreover, the study aimed to verify the association between neurologic outcome and the ROSC-NGAL and 3 h-NGAL levels.

AKI is associated with poor clinical outcome in PCAS patients, and the incidence of AKI in PCAS patients ranges from 12 to 80% [3–7]. In a study by Oh et al. [3] that was published in 2019, AKI occurred in 348 (60%) of 583 patients who underwent TTM and was associated with poor neurologic outcome at 6 months (adjusted OR 0.206; 95% CI 0.099–0.426; *p* < 0.001). In a 2015 study by Geri et al. [4], Stage 3 AKI occurred in 280 out of 580 OHCA patients (48.3%) and was associated with the 30-day mortality rate (OR 1.60; 95% CI 1.05–2.43; *p* = 0.03). A similar tendency was identified in this study, wherein a comparison of survival at discharge, CPC at discharge, and the 1- and 6-month survival in the AKI (+) and AKI (−) groups showed a significantly higher frequency of poor outcome in the AKI (+) group.

The appropriate management of AKI, which has an effect on poor prognosis in PCAS patients, requires the prediction and early management of AKI. In the results reported from a study by Choi et al. [22] in 2020, AKI occurred in 55.5% of 1373 patients who underwent TTM after OHCA, and 78.1% of those patients developed AKI within 3 days after OHCA. Because there is no specific treatment to reverse AKI, early recognition and management are important to improve the clinical outcomes. The recognition of patients at risk for AKI, or those with possible AKI but before the appearance of clinical manifestations, is likely to result in better outcomes than that attained by treating only patients with established AKI [17]. The management of patients at risk for AKI includes the discontinuation of all nephrotoxic agents when possible, ensuring volume status and perfusion pressure, considering functional hemodynamic monitoring, monitoring SCr and urine output, avoiding hyperglycaemia, and considering alternatives to radiocontrast procedures [17]. Moreover, early recognition of AKI is a useful indicator to secure the requisite medical resources, such as CRRT. In the clinical practice guidelines for AKI in 2012, the KDIGO Acute Kidney Injury Work Group emphasised the importance of studies on biomarkers for the early diagnosis, prognosis, and differential diagnosis of AKI [17]. Thus, additional studies are needed on biomarkers for the early diagnosis or risk prediction of AKI, as well as the prediction of mortality or long-term renal replacement therapy in AKI patients. The factors that are being studied as biomarkers for AKI include NGAL, cystatin C, interleukin-18, kidney injury molecule-1, and plasma IL-6. Among the biomarkers of AKI, both NGAL and gamma-glutamyl transpeptidase/alkaline phosphatase have evidence from Phase 4 or higher studies [8].

Human NGAL was originally identified as a novel protein that was isolated from the secondary granules of human neutrophils [23]. Preclinical transcriptome profiling in a number of AKI models revealed NGAL to be one of the most robustly upregulated genes in the kidney post injury [24, 25]. The NGAL has been identified as a useful marker for the early prediction of AKI in situations that confer a potential risk of kidney injury, such as cardiopulmonary bypass, contrast administration, and kidney transplantation [19]. Cardiac surgery-associated AKI is indicated by a more than 10-fold elevation in the urinary and serum levels of NGAL within 2–6 h after surgery. Many prospective studies have reported that patients with AKI showed significantly increased NGAL levels at 1–3 h after surgery [26–29]. Contrast-induced AKI could be predicted by NGAL measurement at 2 h after contrast administration [20, 30–32]. In studies that analysed the association of NGAL in patients with post-OHCA AKI, the timepoint of NGAL varied from immediately after ROSC to several days later, and there were differences in AUROCs or ORs of NGAL for AKI depending on the time of measurement [13–15]. However, no studies have comparatively evaluated NGAL values that were measured immediately after ROSC with those measured hours later. This study focused on the early prediction of AKI, and compared ROSC-NGAL and 3 h-NGAL to determine the clinical utility of NGAL as a predictor of AKI. The results showed that 3 h-NGAL is a more accurate predictor of AKI than ROSC-NGAL. Meanwhile, a study has been published recently which refuted prevailing opinion that NGAL is produced in the kidneys. A study by Skrypnyk et al. showed that an increase in IL-6 in wild-type mice with ischemic AKI induces the hepatic production of NGAL, thereby increasing the plasma NGAL levels. Based on their result, they predicted that NGAL levels may increase regardless of whether AKI occurs in conditions wherein plasma IL-6 levels can increase. Those authors reported the need for further studies to validate this result with regard to AKI in the general [12].

Research has been actively undertaken on NGAL and clinical outcomes in OHCA patients. In a 2019 study by Lee et al. [33], the plasma NGAL measured 4 h after ROSC among adult OHCA patients who were treated with TTM was associated with both the neurologic outcome at the time of discharge (adjusted OR 1.004; 95% CI 1.001–1.007) as well as the 28-day mortality rate (adjusted OR 1.003; 95% CI 1.001–1.004). In 2018, Park et al. [34] reported that the NGAL level was measured immediately and 24, 46, and 72 h after ROSC and was analysed to predict the long-term outcome and survival in 76 OHCA patients who underwent TTM; the results showed that the NGAL value measured after 72 h was the optimal predictive indicator for the outcome and survival (AUROC = 0.72; *p* = 0.02). In a 2017 study, Kaneko et al. [35] analysed the neurologic outcome at discharge based on NGAL measurements at 1 and 2 days after ROSC; the NGAL level after 2 days showed a comparable predictive value as the 2-day neuron-specific enolase, which has widespread application in the prediction of the neurologic outcome. In this study, the results of multivariate regression analysis of ROSC-NGAL and 3 h-NGAL measurements did not identify them as significant risk factors for poor neurologic outcome (ROSC-NGAL: OR 1.017; 95% CI 0.998–1.036; *p* = 0.084, 3 h-NGAL: OR 0.997; 95% CI 0.992–1.001; *p* = 0.113). The neuroprognostic value of NGAL measured within 24 h is remains controversial [34]; therefore, additional studies are necessary to determine the optimal timepoints for NGAL measurement after ROSC.

This study had several limitations. First, in patients with missing data for the serum creatinine level, we used the creatinine level on the first day of hospitalisation as the baseline value. Second, the study did not consider the potential effect of the history of concurrent medications and the radiocontrast procedure on renal function, volume status after admission, and the onset of complications. Third, the single-centre study design is another limitation of this study. Forth, number of study patients was low. The frequency of OHCA is extremely low, and this limitation is difficult to avoid due to the nature of the disease. However, this drawback may be circumvented by multicentre research studies. Fourth, a recent study reported the hepatic production of NGAL via the IL-6 level. Therefore, the results of this study should be interpreted with due consideration to conditions that may increase IL-6 as well as the risk of AKI.

## Conclusions

The serum NGAL concentration measured at 3 h after ROSC is an excellent early predictive marker for AKI in OHCA patients treated with TTM. Further research is needed to identify the optimal timepoint of measurement to establish NGAL as a predictor of the neurologic outcome and to validate the findings of this research.

## Supplementary information

**Additional file 1.** General and clinical characteristics of study patients according to AKI stages

## Data Availability

The datasets used and/or analysed during the current study are available from the corresponding author on reasonable request.

## Abbreviations

NGAL: Neutrophil gelatinase-associated lipocalin
AKI: Acute kidney injury
ROSC: Return of spontaneous circulation
OHCA: Out-of-hospital cardiac arrest
TTM: Targeted temperature management
PCAS: Post-cardiac arrest syndrome
IL-6: Interleukin-6
KDIGO: Kidney Disease Improving Global Outcomes
SCr: Serum creatinine
CPC: Cerebral performance category
GNO: Good neurologic outcome
PNO: Poor neurologic outcome
CPR: Cardiopulmonary resuscitation
EMS: Emergency medical service
OR: Odds ratio
CI: Confidence interval
ROC: Receiver operating characteristics
AUROC: Area under the receiver operating characteristics curve
Vf: Ventricular fibrillation
PEA: Pulseless electrical activity
