# Supplementary material for: The title: serum neutrophil Gelatinase-associated Lipocalin at 3 hours after return of spontaneous circulation in patients with cardiac arrest and therapeutic hypothermia: early predictor of acute kidney injury
Source: BMC Nephrol. 2020 Sep 7;21:389. doi: 10.1186/s12882-020-02054-7 (PMC7487645; doi:10.1186/s12882-020-02054-7)
Supplement: Supplementary file 1 — Additional file 1. General and clinical characteristics of study patients according to AKI stages [file 12882_2020_2054_MOESM1_ESM.docx]

Additional file 1. General and clinical characteristics of study patients according to AKI stage

| Characteristics | Total | | AKI | | | | | | | | |
| --- | --- | --- | --- | --- | --- | --- | --- | --- | --- | --- | --- |
|  |  |  | (-) | | 1 | | 2 | | 3 | | *p*-value |
| Number of patients | 89 | (100.0) | 40 | (44.9) | 21 | (23.6) | 12 | (13.5) | 16 | (18.0) |  |
|  |  |  |  |  |  |  |  |  |  |  |  |
| Sex |  |  |  |  |  |  |  |  |  |  | 0.500 |
| Male | 63 | (70.8) | 30 | (75.0) | 16 | (76.2) | 8 | (66.7) | 9 | (56.3) |  |
| Female | 26 | (29.2) | 10 | (25.0) | 5 | (23.8) | 4 | (33.3) | 7 | (43.8) |  |
|  |  |  |  |  |  |  |  |  |  |  |  |
| Age (years) | 56.0 | (43.5-66.0) | 53.0 | (43.0-62.0) | 56.0 | (35.5-59.5) | 61.5 | (49.5-72.0) | 61.0 | (46.8-68.5) | 0.166 |
|  |  |  |  |  |  |  |  |  |  |  |  |
| Medical history |  |  |  |  |  |  |  |  |  |  |  |
| HTN | 28 | (31.5) | 11 | (27.5) | 5 | (23.8) | 5 | (41.7) | 7 | (43.8) | 0.464 |
| DM | 18 | (20.2) | 5 | (12.5) | 2 | (9.5) | 5 | (41.7) | 6 | (37.5) | **0.025** |
| HF | 2 | (2.2) | 1 | (2.5) | 0 | (0.0) | 0 | (0.0) | 1 | (6.3) | 0.586 |
|  |  |  |  |  |  |  |  |  |  |  |  |
| Initial rhythm by EMS or hospital |  |  |  |  |  |  |  |  |  |  | **0.001** |
| Vf | 40 | (44.9) | 26 | (65.0) | 5 | (23.8) | 3 | (25.0) | 6 | (37.5) |  |
| PEA | 18 | (20.2) | 9 | (22.5) | 4 | (19.0) | 3 | (25.0) | 2 | (12.5) |  |
| Asystol | 28 | (31.5) | 4 | (10.0) | 12 | (57.1) | 6 | (50.0) | 6 | (37.5) |  |
| Unknown | 3 | (3.4) | 1 | (2.5) | 0 | (0.0) | 0 | (0.0) | 2 | (12.5) |  |
|  |  |  |  |  |  |  |  |  |  |  |  |
| Witness cardiac arrest | 66 | (74.2) | 37 | (92.5) | 10 | (47.6) | 7 | (58.3) | 12 | (75.0) | **0.001** |
| Bystander CPR | 49 | (55.1) | 25 | (62.5) | 12 | (57.1) | 3 | (25.0) | 9 | (56.3) | 0.150 |
| EMS activation to EMS arrival (min) | 7.0 | (5.6-10.0) | 7.0 | (6.0-9.0) | 6.0 | (5.0-10.8) | 8.0 | (4.5-11.5) | 7.5 | (5.3-9.5) | 0.994 |
| EMS activation to fisrt defibrillation (min) | 7.5 | (6.0-12.0) | 7.5 | (6.0-10.0) | 8.0 | (6.0-14.5) | 8.0 | (4.75-14.8) | 7.5 | (6.0-11.5) | 0.987 |
| Time to ROSC (min) | 25.0 | (13.8-35.0) | 19.0 | (11.5-33.5) | 16.0 | (13.0-34.0) | 31.0 | (14.0-37.0) | 33.0 | (15.0-39.0) | 0.327 |
| Epinephrine dose during CPR | 1.0 | (0.0-3.0) | 0.0 | (0.0-1.0) | 2.0 | (0.5-3.0) | 2.5 | (2.0-8.5) | 4.0 | (2.0-4.0) | **<0.001** |
|  |  |  |  |  |  |  |  |  |  |  |  |
| Baseline creatinine level | 1.01 | (0.89-1.24) | 1.01 | (0.89-1.26) | 1.16 | (1.00-1.39) | 0.92 | (0.85-1.05) | 0.99 | (0.80-1.21) | 0.050 |
| Peak creatinine level | 1.35 | (1.13-2.25) | 1.14 | (1.04-1.26) | 1.35 | (1.13-1.50) | 2.11 | (1.99-2.38) | 4.66 | (3.70-7.38) | **<0.001** |
|  |  |  |  |  |  |  |  |  |  |  |  |
| NGAL at ROSC | 124.0 | (96.0-186.0) | 105.5 | (83.3-143.3) | 126.0 | (101.0-155.5) | 162.5 | (88.0-270.5) | 177.5 | (120.0-76.5) | **0.003** |
| NGAL at 3h after ROSC | 181.0 | (115.0-381.0) | 115.0 | (84.3-145.5) | 258.0 | (182.0-385.0) | 370.5 | (239.3-685.5) | 588.5 | (271.8-2032.0) | **<0.001** |
|  |  |  |  |  |  |  |  |  |  |  |  |
| CRRT | 11 | (14.7) | 0 | (0.0) | 1 | (4.8) | 0 | (0.0) | 10 | (62.5) | **<0.001** |
| CAG | 40 | (44.9) | 25 | (62.5) | 8 | (38.1) | 2 | (16.7) | 5 | (31.3) | **0.015** |
| Target temperature |  |  |  |  |  |  |  |  |  |  | 0.737 |
| 33℃ | 86 | (96.6) | 38 | (95.0) | 21 | (100.0) | 12 | (100.0) | 15 | (93.8) |  |
| <36℃ | 3 | (3.4) | 2 | (5.0) | 0 | (0.0) | 0 | (0.0) | 1 | (6.3) |  |
| TTM duration |  |  |  |  |  |  |  |  |  |  | 0.364 |
| 24h | 85 | (95.5) | 39 | (97.5) | 20 | (95.2) | 12 | (100.0) | 14 | (87.5) |  |
| 48h | 4 | (4.5) | 1 | (2.5) | 1 | (4.8) | 0 | (0.0) | 2 | (12.5) |  |
| Survival discharge | 48 | (53.9) | 34 | (85.0) | 9 | (42.9) | 3 | (25.0) | 2 | (12.5) | **<0.001** |
| CPC at discharge |  |  |  |  |  |  |  |  |  |  | **<0.001** |
| Good (1,2) | 35 | (39.3) | 26 | (65.0) | 6 | (28.6) | 2 | (16.7) | 1 | (6.3) |  |
| Poor (3,4,5) | 54 | (60.7) | 14 | (35.0) | 15 | (71.4) | 10 | (83.3) | 15 | (93.8) |  |
| Survival at 1 month | 47 | (52.8) | 33 | (82.5) | 9 | (42.9) | 2 | (16.7) | 3 | (18.8) | **<0.001** |
| Survival at 6 months | 47 | (53.4) | 33 | (82.5) | 9 | (42.9) | 2 | (18.2) | 3 | (18.8) | **<0.001** |

Quantitative data are expressed as median (interquartile range), categorical data are presented as number of subjects (percentages). Kruskal-Wallis test was used for continuous variable analysis, while chi-squared test or Fisher’s exact test were used for categorical variable analysis as appropriate

*AKI* acute kidney injury, *HTN* hypertension, *DM* diabetes mellitus, *HF* heart failure, *Vf* ventricular fibrillation, *PEA* pulseless electrical activity, *CPR* cardiopulmonary resuscitation, *EMS* emergency medical system, *ROSC* return of spontaneous circulation, *NGAL* neutrophil gelatinase-associated lipocalin, *ROSC* return of spontaneous circulation, *CRRT* continuous renal replacement therapy, *CAG* coronary angiography, *TTM* targeted temperature management, *CPC* cerebral performance category
